# Up-regulation of claudin-2 expression by aldosterone in colonic epithelial cells of mice fed with NaCl-depleted diets

**DOI:** 10.1038/s41598-017-12494-1

**Published:** 2017-09-22

**Authors:** Chisa Furukawa, Noriko Ishizuka, Hisayoshi Hayashi, Naoko Fujii, Aya Manabe, Yoshiaki Tabuchi, Toshiyuki Matsunaga, Satoshi Endo, Akira Ikari

**Affiliations:** 1 Laboratory of Biochemistry, Department of Biopharmaceutical Sciences, Gifu Pharmaceutical University, Gifu, Japan; 2 School of Food and Nutritional Sciences, University of Shizuoka, Shizuoka, Japan; 3 Life Science Research Center, University of Toyama, Toyama, Japan

## Abstract

Dietary NaCl depletion increases Na^+^ absorption and K^+^ secretion in the colon, but the mechanisms are not fully understood. In mice fed with NaCl-depleted diets, the expression of claudin-2 and -7 increased compared to those in control mice. Aldosterone (ALD) concentration was also increased. We examined the regulatory mechanism of claudin expression by ALD using the murine colonic epithelial MCE301 cells. ALD dose-dependently increased claudin-2 expression without affecting the expression of claudin-4, -7, -8, and -15. ALD increased nuclear distribution of mineralocorticoid receptor (MR), which was inhibited by spironolactone, an MR antagonist. The ALD-induced elevation of claudin-2 mRNA and protein expression was inhibited by spironolactone, but not by RU-486, a glucocorticoid receptor antagonist. Luciferase reporter assay showed that ALD interacts with the promoter region between -2,021 and -2,008 of human claudin-2. The binding of MR on the promoter region of claudin-2 was increased by ALD, which was inhibited by spironolactone in chromatin immunoprecipitation assay. Our data suggest that ALD acts on MR and increases paracellular permeability to ions mediated by the elevation of claudin-2 expression in the colon. NaCl depletion may increase ALD secretion from adrenal cortex, resulting in the elevation of paracellular permeability to cations in the colon.

## Introduction

The net flux rates of Na^+^, K^+^, Cl^−^ and HCO_3_^−^ in the colon vary according to the concentration in the lumen^1^. Plasma levels of renin and aldosterone (ALD) are elevated during a low Na^+^ intake or Na^+^ depletion^2^. The secretion of ALD is primarily controlled by the renal renin-angiotensin system and is reduced by bilateral nephrectomy^3^. Mineralocorticoid receptor (MR) is expressed in the surface epithelium and crypts of proximal and distal colon^4^. ALD increases Na^+^ absorption in both proximal and distal segments, but the mechanism of ALD is different in these segments^5^. ALD increases amiloride-insensitive Na^+^ absorption accompanied by unchangeable potential difference and slight elevation of short circuit current (Isc) in proximal colon. In contrast, ALD increases amiloride-sensitive Na^+^ absorption accompanied by an elevation of transmural potential and Isc in distal colon. Na^+^ transport may occur through electroneutral absorption by luminal Na^+^/H^+^-exchanger type 3 (NHE3) in proximal colon and electrogenic epithelial Na^+^ channel (ENaC), which is inhibited by amiloride, in distal colon^6^. Thus, the transcellular Na^+^ absorption is well characterized, but the mechanism that controls paracellular shunt is poorly understood.

At the apical pole of the intercellular junction of the lateral membrane, epithelial cells form the tight junctions (TJs), which compose a large complex of proteins including the membrane integral proteins such as claudins, occludin and tricellulin, and the scaffolding proteins such as zonula occludens (ZO)-1, -2, and -3^7^. The TJs separate the apical and basolateral epithelial compartments to produce their polarization and create a primary barrier to control the diffusion of solutes across the epithelial sheet. Claudins are the most important structural and functional components of the TJs. They comprise a family of over 20 members and bear common structure of four transmembrane domains with a short cytoplasmic N-terminus, two extracellular loops (ECLs) and a C-terminal cytoplasmic domain^8,9^. The first ECL containing several negatively or positively charged amino acids may be critical for determining the paracellular ion permeability and the second ECL contributes to homo- and/or heterophilic trans-interaction of claudins. Different combinations of claudins can confer different paracellular permeability to epithelial cells^10^.

The epithelial cells of gastrointestinal tract in mice highly express claudin-2, -3, -7, and -15, while weakly express claudin-1, -5, -8, -9, -10, and -11. A segment specific expression pattern of claudins determines the tightness of the TJs from proximal to distal segments. Both claudin-2 and -15 forms a cation permeable pore, but the expression pattern is different. Claudin-2 is expressed in the deep crypt of distal colon, whereas claudin-15 is expressed in the small intestinal villi and in colonic surface cells. The different expression pattern of these claudins is also observed during postnatal development. Claudin-2 expression diminishes progressively after birth, whereas claudin-15 expression increases 10-fold between 14 and 28 days^11^. To understand how cations homeostasis is maintained under the physiological and pathophysiological conditions, we have to investigate the regulatory factor and mechanism of colonic claudin-2 and -15 expression.

In the present study, we found that NaCl-depleted diets increases serum ALD concentration and colonic expression of claudin-2 and -7 in mice. ALD increased the expression of claudin-2, which was blocked by spironolactone, an MR antagonist, in the murine colonic epithelial MCE301 cells. ALD decreased transepithelial electrical resistance (TER), which was also blocked by spironolactone. NaCl depletion increased the abundance of claudin2 in the distal colon. Up-regulation of claudin-2 may be involved in the ALD-induced cation transport in the colon.

## Results

### Increase in claudin-2 and -7 expression by NaCl depletion

**Figure 1:**
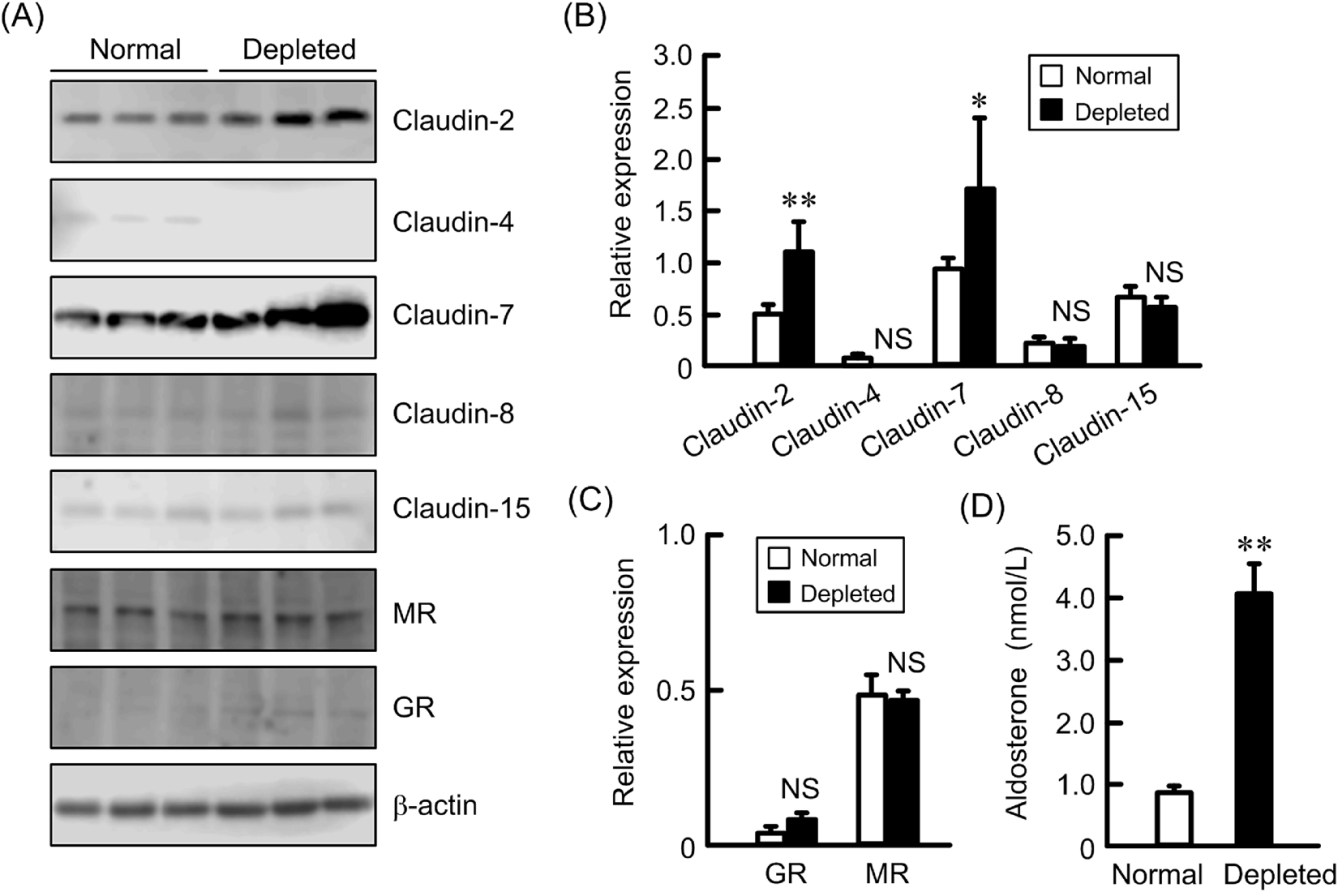
Effect of NaCl depletion on colonic claudins expression and serum ALD concentration. (**A**) The distal colon was isolated from the mice fed normal and NaCl-depleted diets. Cytoplasmic extracts including membrane and cytoplasmic proteins of colon were prepared from three independent preparations and immunoblotted with anti-claudin-2, -4, -7, -8, -15, MR, GR, or β-actin antibody. (**B**) The contents of claudins were represented relative to the values of β-actin. (**C**) The contents of MR and GR were represented relative to the values of β-actin. (**D**) Serum ALD concentrations in the mice fed normal and NaCl-depleted diets were measured. n = 3. Statistical comparison was made by *t* test. **P < 0.01 and *P < 0.05 significantly different from normal. NS, not significantly different.

Mice were fed ad libitum diets containing normal and depleted NaCl for 10 days. NaCl depletion significantly increased the protein levels of claudin-2 and -7, whereas it did not change those of claudin-4, -8, and -15, MR, and glucocorticoid receptor (GR) in the colon (Fig. 1A–C). Serum ALD concentration was significantly increased by NaCl depletion (Fig. 1D). These results raised a possibility that ALD is involved in the up-regulation of claudin-2 and -7 in the colon. Therefore, we examined the effect of ALD on claudin-2 and -7 expression using murine colonic epithelial MCE301 cells.

### Effects of culture periods on claudins expression and paracellular permeability in MCE301 cells

**Figure 2:**
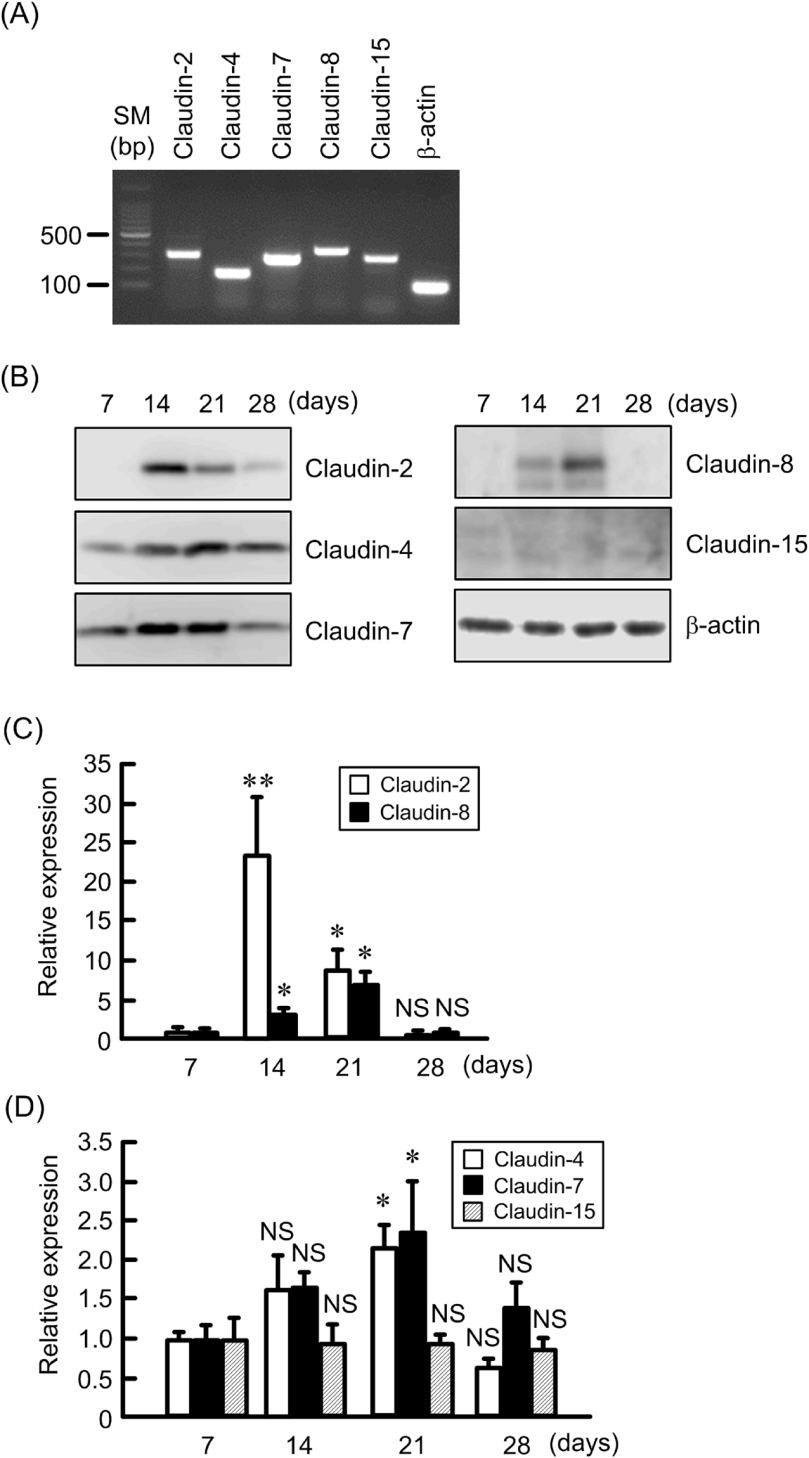
Effects of culture periods on claudins expression in MCE301 cells. (**A**) MCE301 cells were cultured for 21 days. After isolation of total RNA, RT-PCR was performed using primers pair of mouse claudin-2, -4, -7, -8, -15, and β-actin. The size marker (SM) is indicated at the left. (**B**) Cells were culture for the periods indicated. Cytoplasmic extracts including membrane and cytoplasmic proteins were prepared from four independent preparations and immunoblotted with anti-claudin-2, -4, -7, -8, -15, or β-actin antibody. (**C** and **D**) The contents of claudins were represented relative to the values at 7 days. Statistical comparison was made by *t* test. **P < 0.01 and *P < 0.05 significantly different from 7 days. NS, not significantly different.

**Figure 3:**
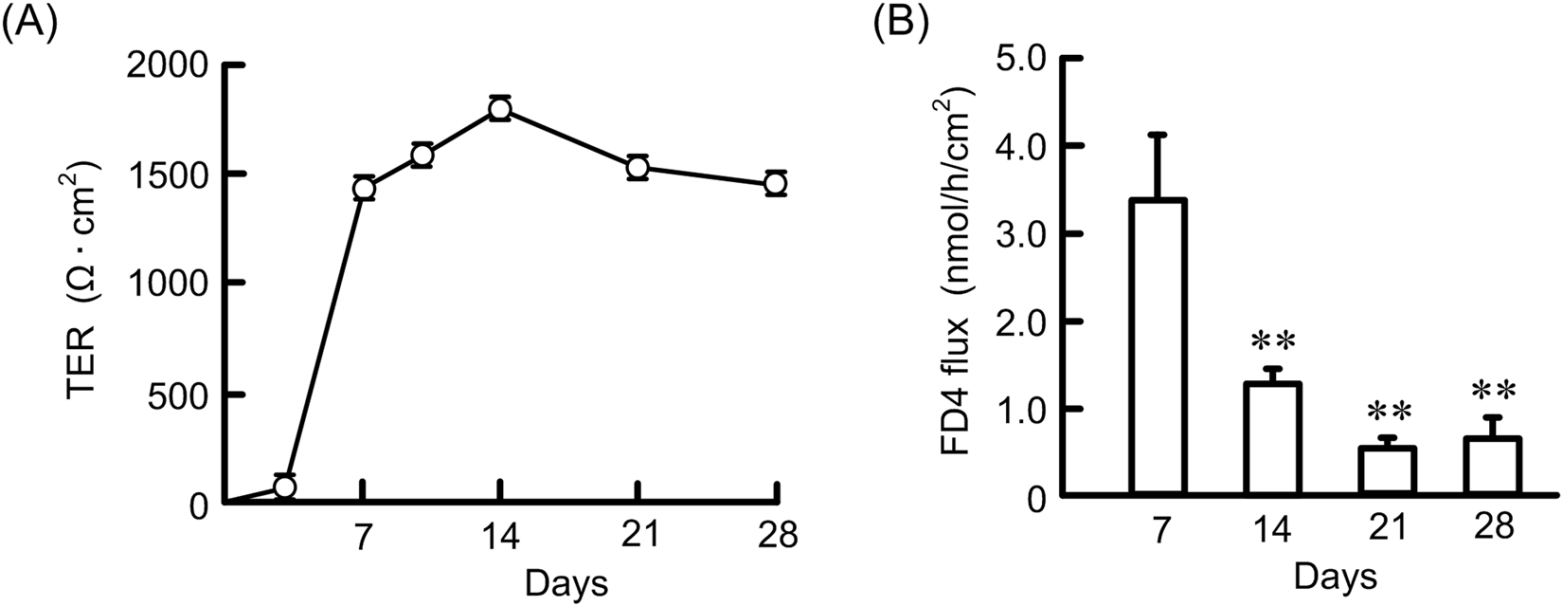
Effects of culture periods on paracellular permeability. (**A**) Cells were cultured on transwells for the periods indicated. TER was measured using volt-ohmmeter. (**B**) FD4 was applied to the apical compartment. After 1 h, the buffer in the opposite compartment was collected and fluorescence intensity was measured. n = 4. **P < 0.01 significantly different from 7 days.

RT-PCR showed that the mRNAs of claudin-2, -4, -7, -8, and -15 are expressed in MCE301 cells cultured for 21 days (Fig. 2A). The protein levels of claudin-2, -4, -7, and -8 time-dependently increased and reached a peak at 14 or 21 days, but the expression of claudin-15 was at a constant low level (Fig. 2B–D). Paracellular permeability of MCE301 cells was estimated by TER and fluorescein isothiocyanate-dextran 4 kDa (FD4) flux. TER time-dependently increased and reached a peak at 14 days, followed by a plateau phase above baseline (Fig. 3). On the contrary, FD4 flux time-dependently decreased and reached a plateau phase at 14 days. These results indicate that MCE301 cells may form functional TJs after 14 days.

### Increase in claudin-2 expression by ALD

**Figure 4:**
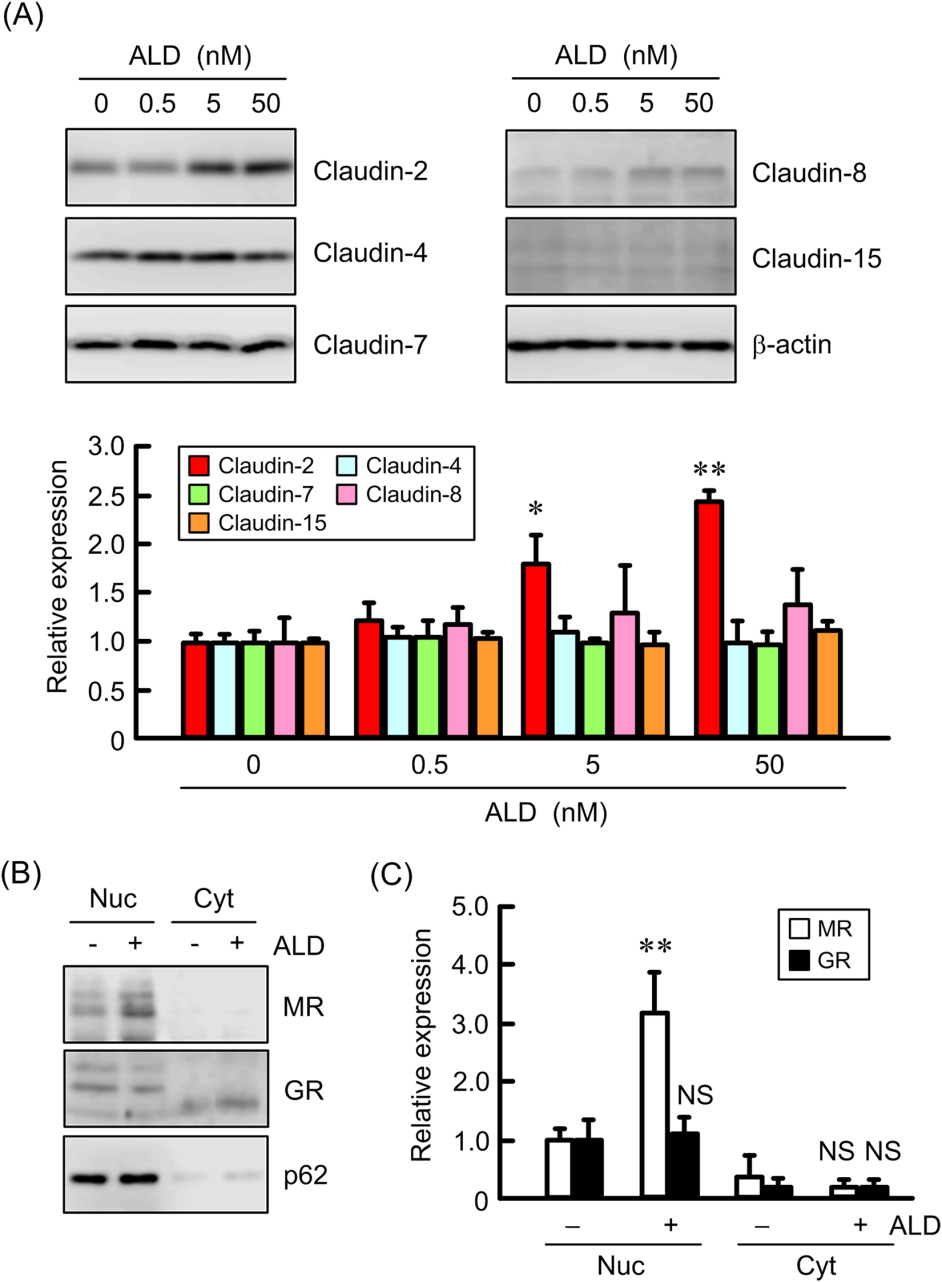
Increase in claudin-2 expression by ALD in MCE301 cells. (**A**) Cells were treated with ALD for 24 h in the concentration indicated. Cytoplasmic extracts including membrane and cytoplasmic proteins were prepared from four independent preparations and immunoblotted with anti-claudin-2, -4, -7, -8, -15, or β-actin antibody. The contents of claudins were represented relative to the values in 0 nM. (**B**) The cells were treated with 50 nM ALD for 2 h. Nuclear (Nuc) and cytoplasmic (Cyt) fractions were prepared from three independent preparations and immunoblotted with anti-MR, GR, or nucleoporin p62 antibody. (**C**) The contents of MR and GR were represented relative to the values of nuclear fraction in the absence of ALD. **P < 0.01 and *P < 0.05 significantly different from 0 nM. NS, not significantly different from - ALD.

**Figure 5:**
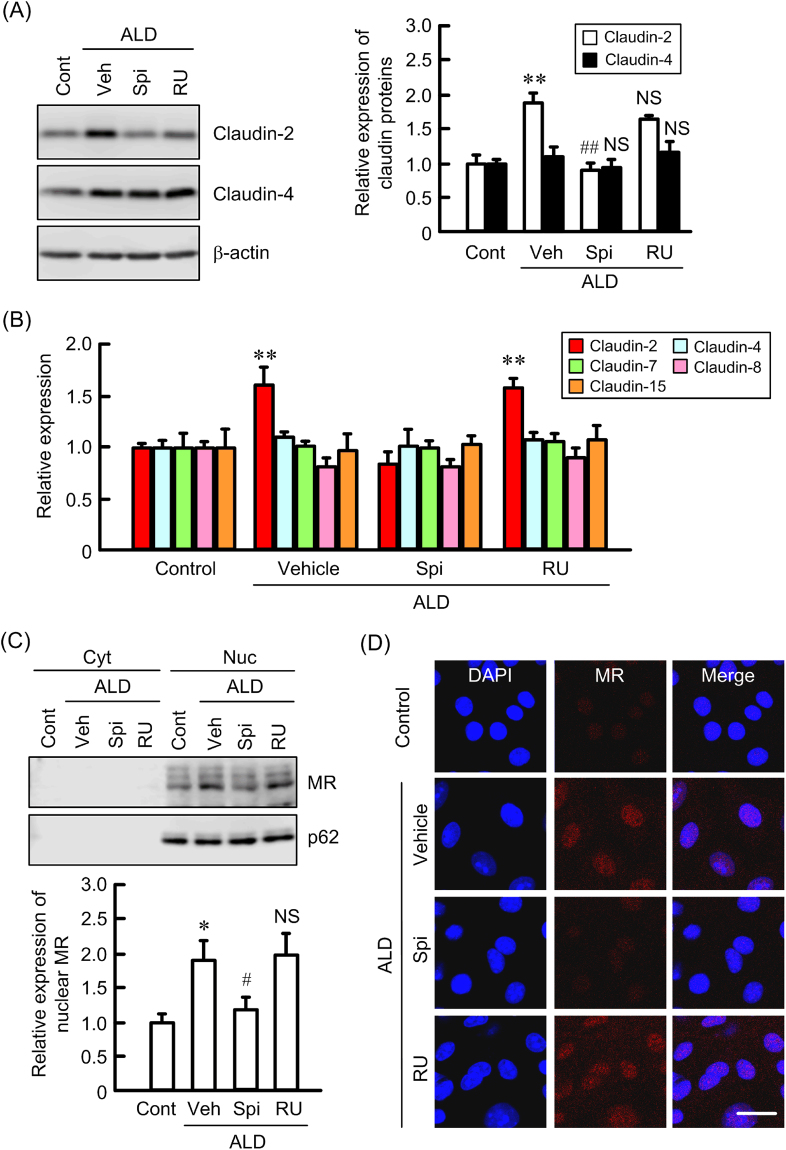
Inhibition of ALD-induced increase in claudin-2 expression by spironolactone. (**A**) Cells were treated with 50 nM ALD for 24 h in the presence and absence of 10 μM spironolactone (Spi) or 10 μM RU-486 (RU). Control cells (Cont) were not treated with these drugs. Vehicle (Veh) was treated with dimethylsulfoxide. Cytoplasmic extracts including membrane and cytoplasmic proteins were prepared from three independent preparations and immunoblotted with anti-claudin-2, -4, or β-actin antibody. The contents of claudins were represented relative to the values in control. (**B**) After isolation of total RNA, real-time RT-PCR was performed using primers pair of mouse claudin-2, -4, and β-actin. (**C**) The cells were treated with ALD for 2 h in the presence and absence of Spi or RU. Nuclear (Nuc) and cytoplasmic (Cyt) fractions were prepared from four independent preparations and immunoblotted with anti-MR or nucleoporin p62 antibody. (**D**) Cells were stained with MR (red) and DAPI (blue). The scale bar represents 10 μm. **P < 0.01 and *P < 0.05 significantly different from control. ^##^P < 0.01 significantly different from vehicle. NS, not significantly different from vehicle.

ALD dose-dependently increased the protein levels of claudin-2 without affecting those of claudin-4, -7, -8, and -15 (Fig. 4A). ALD also increased the nuclear distribution of MR, but not that of GR (Fig. 4B and C). Nucleoporin p62 served as an internal control of the nuclei. These results indicate that the activation of MR may be involved in the elevation of claudin-2 by ALD. Therefore, we examined the effects of inhibitors of MR and GR on claudin-2 expression. The ALD-induced elevation of claudin-2 protein was significantly inhibited by spironolactone, an MR antagonist. RU-486, a GR antagonist, slightly inhibited the ALD-induced elevation of claudin-2 protein, but the effect was lower than that of spironolactone (Fig. 5A). Similarly, the ALD-induced elevation of claudin-2 mRNA was significantly inhibited by spironolactone (Fig. 5B), but not by RU-486. Furthermore, sub-fractionated western blotting and immunofluorescence measurement showed that the nuclear distribution of MR induced by ALD is inhibited by spironolactone, but not by RU-486 (Fig. 5C and D). These results indicate that ALD may increase claudin-2 expression mediated by the elevation of nuclear distribution of MR.

### Increase in promoter activity of claudin-2 and binding of MR on the promoter by ALD

**Figure 6:**
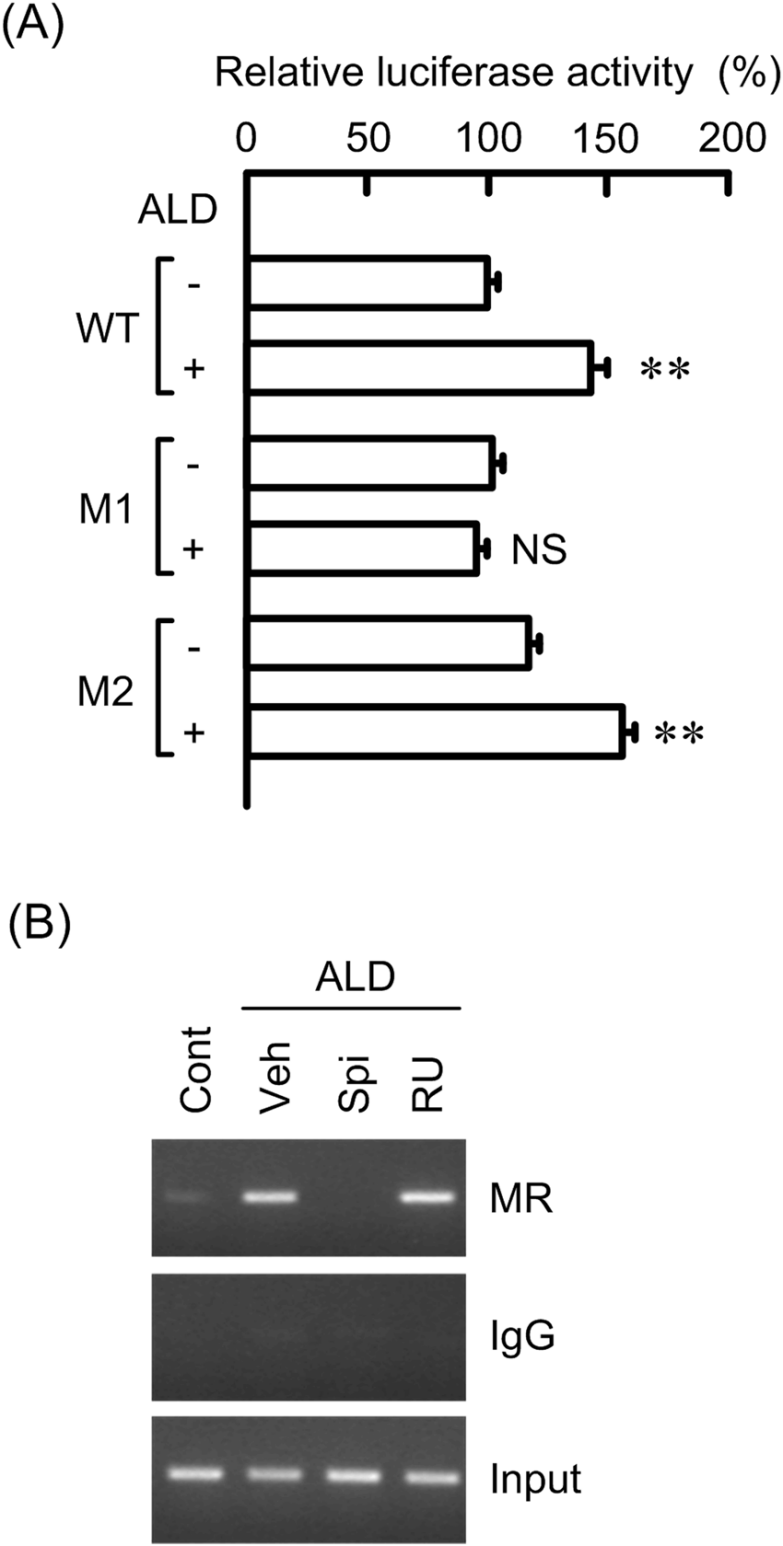
Binding of MR to claudin-2 promoter by ALD. (**A**) Promoter luciferase constructs of claudin-2 including wild type (WT), mutant of SRE-1 (M1), and mutant of SRE-2 (M2) were co-transfected with pRL-TK vector into the cells. After 40 h of transfection, the cells were incubated in the presence and absence of 50 nM ALD for additional 8 h. The relative promoter activity was represented as the percent of WT in the absence of ALD. (**B**) Nuclear proteins were prepared from the cells treated with 50 nM ALD for 1 h in the presence and absence of 10 μM spironolactone (Spi) or 10 μM RU-486 (RU). Control cells (Cont) were not treated with these drugs. Vehicle (Veh) was treated with dimethylsulfoxide. After immunoprecipitation of genomic DNA by anti-MR antibody or rabbit IgG, semi-quantitative PCR was performed using the primers amplifying the MR binding site of claudin-2 promoter. Input chromatin was used for loading control. Statistical comparison was made by *t* test. **P < 0.01 significantly different from - ALD. NS, not significantly different from - ALD.

Two presumable sites of MR action was detected in the promoter region of human claudin-2 (Supplementary Figure S1). ALD significantly increased the promoter activity of claudin-2 (Fig. 6A). The ALD-induced promoter activity was not inhibited in the mutant 2, which contains mutation in steroid response element (SRE)-2 of promoter region, whereas it was inhibited in the mutant 1, which contains mutation in SRE-1. These results indicate that MR may interact to the region between -2,021 and -2,008 within the promoter of claudin-2. In the chromatin immunoprecipitation (ChIP) assay, the binding of MR on the promoter region of claudin-2 was increased by ALD, which was inhibited by spironolactone, but not by RU-486 (Fig. 6B). These results are similar to those in Western blotting and real-time PCR.

### Elevation of tight junctional localization of claudin-2 and paracellular permeability

**Figure 7:**
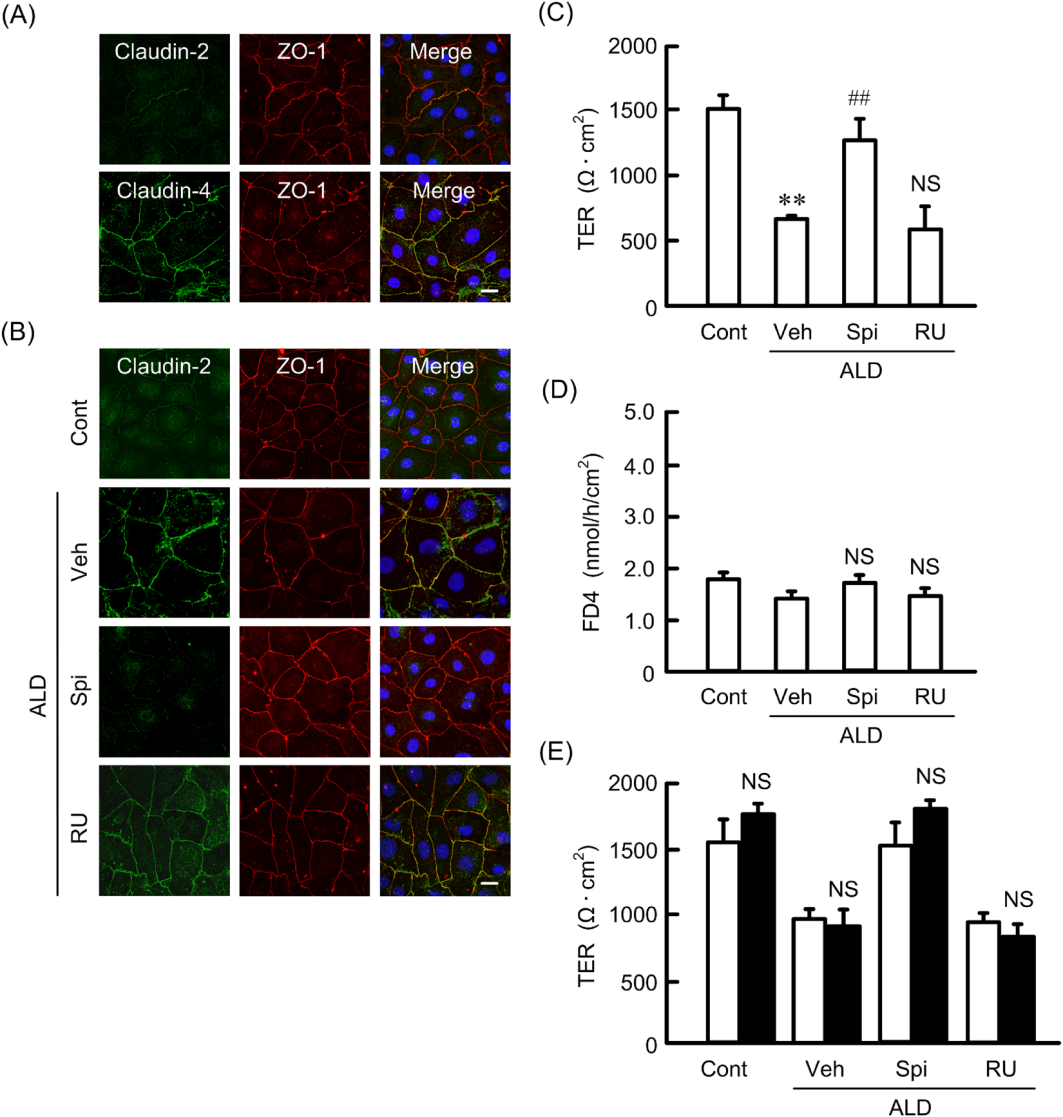
Effects of ALD and spironolactone on the intracellular localization and function of claudin-2. (**A**) Cells were stained with claudin-2 or -4 (green) and ZO-1 (red). Merged images with DAPI staining of the nuclei are shown on the right. (**B**) Cells were treated with 50 nM ALD for 24 h in the presence and absence of 10 μM spironolactone (Spi) or 10 μM RU-486 (RU). Control cells (Cont) were not treated with these drugs. Vehicle (Veh) was treated with dimethylsulfoxide. The cells were stained with claudin-2 (green) and ZO-1 (red). The scale bar represents 20 μm. (**C** and **E**) Cells were cultured on transwell inserts for 21 days. Then the cells were incubated with 50 nM ALD in the presence and absence of 10 μM spironolactone (Spi) or 10 μM RU-486 (RU) for 24 h. TER was measured with volt ohmmeter (**C**). FD4 was applied to the apical compartment. The buffer in the basal compartment was collected after 1 h, and fluorescence intensity was measured (**D**). The cells were incubated with 50 μM amiloride (solid bars) or without amiloride (open bars) for 30 min, followed by measurement of TER. n = 6. Statistical comparison was made by Tukey’s test. **P < 0.01 and ^##^ *P* < 0.01 significantly different from control and ALD, respectively. NS, not significantly different from vehicle.

We examined the intracellular localization of claudin-2 and -4 by immunofluorescence measurements. Under the control conditions, claudin-4 was localized at the TJs concomitant with zonula occludens-1 (ZO-1), a tight junctional scaffolding protein (Fig. 7A). In contrast, the expression level of claudin-2 at the TJs was very low. ALD increased the localization of claudin-2 at the TJs, which was inhibited by spironolactone, but not by RU-486 (Fig. 7B). These results indicate that ALD increases the tight junctional localization of claudin-2 mediated by the activation of MR. Next, we estimated the effect of ALD on paracellular permeability by TER and FD4 flux. ALD significantly decreased TER, which was inhibited by spironolactone, but not by RU-486 (Fig. 7C). These results are similar to those in real-time PCR, western blotting, and immunofluorescence measurements. In contrast, FD4 flux was unchanged by ALD, spironolactone, and RU-486 (Fig. 7D). These results indicate that ALD increases paracellular permeability to ions without affecting small solutes flux. To clarify whether ENaC is involved in the reduction of TER by ALD, we examined the effect of amiloride on TER. The basal and ALD-induced reduction of TER were not significantly blocked by amiloride (Fig. 7E). These results indicate that TER may reflect changes in the characteristics of the paracellular pathway.

### Elevation of claudin-2 expression in distal colon by NaCl depletion

**Figure 8:**
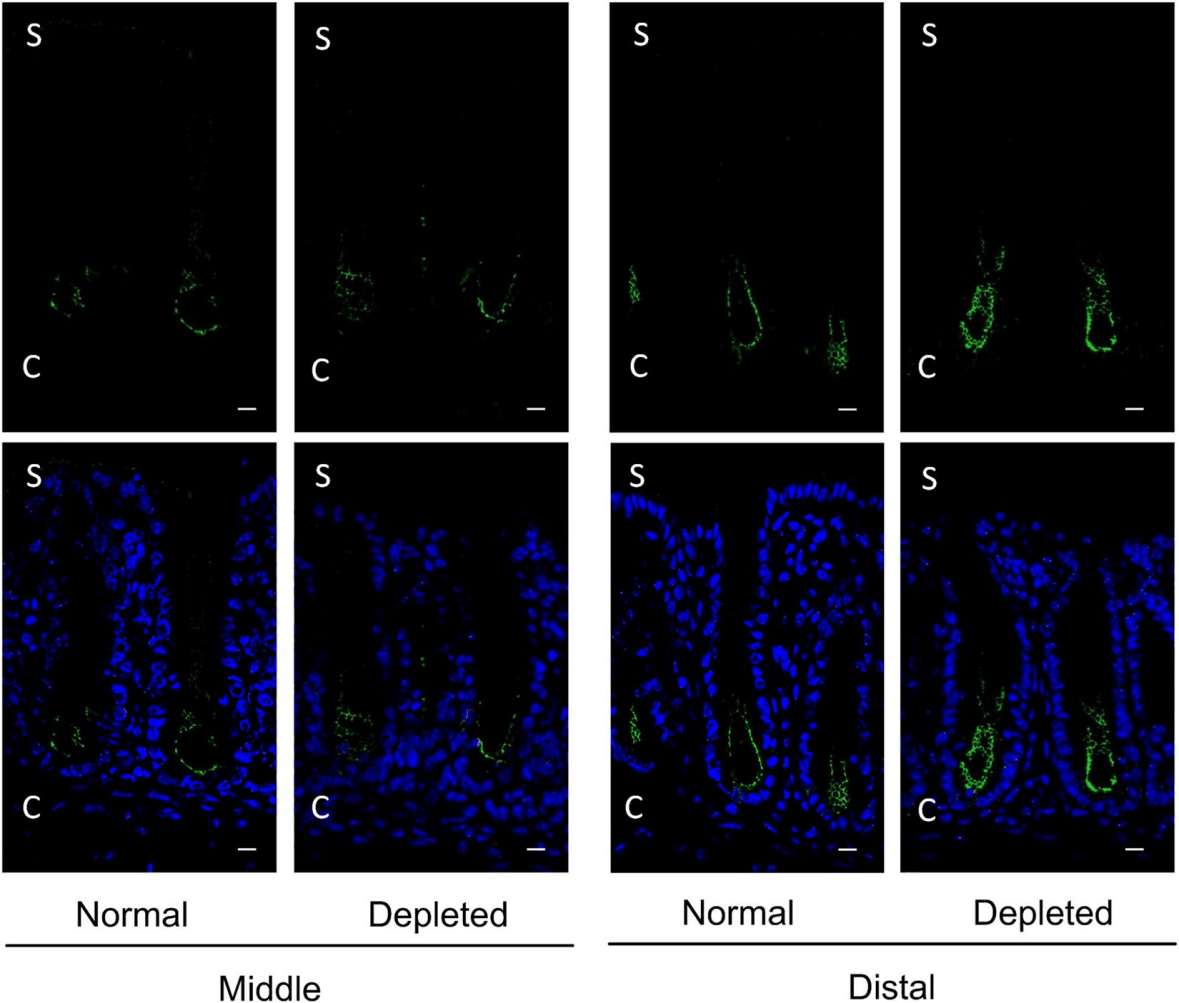
Relative images of immunohistochemical localization of claudin 2 in the mouse colon. The segments of mouse middle and distal colon were isolated from the mice fed normal (n = 3) and NaCl-depleted diets (n = 4). They were stained with claudin-2 (green) and shown on the upper half. Merged images with DAPI staining of the nuclei are shown on the lower half. S and C represent surface and crypt, respectively. The scale bar represents 10 μm.

As described above, ALD should be involved in the up-regulation of claudin-2 in the colon in the mice fed with NaCl-depleted diets. To clarify the segments of elevation of claudin-2 expression, we conducted immunofluorescence experiments in the middle and distal colon. NaCl depletion resulted in changing the localization and increment of abundance of claudin2 in the distal colon, but not middle colon (Fig. 8). Claudin-2 was exclusively localized at the lower one-third of the crypt epithelial cells in the middle and distal colon under normal diets. No specific labeling of surface cells was identified. Dietary NaCl depletion resulted in an increase in intensity of fluorescence and expansion of fluorescence staining to middle part of crypt in the distal colon. In contrast, there was no discernable change in immunofluorescence in the middle colon.

### Effect of NaCl depletion on the expression of Na^+^, K^+^, and H^+^ transport systems

**Figure 9:**
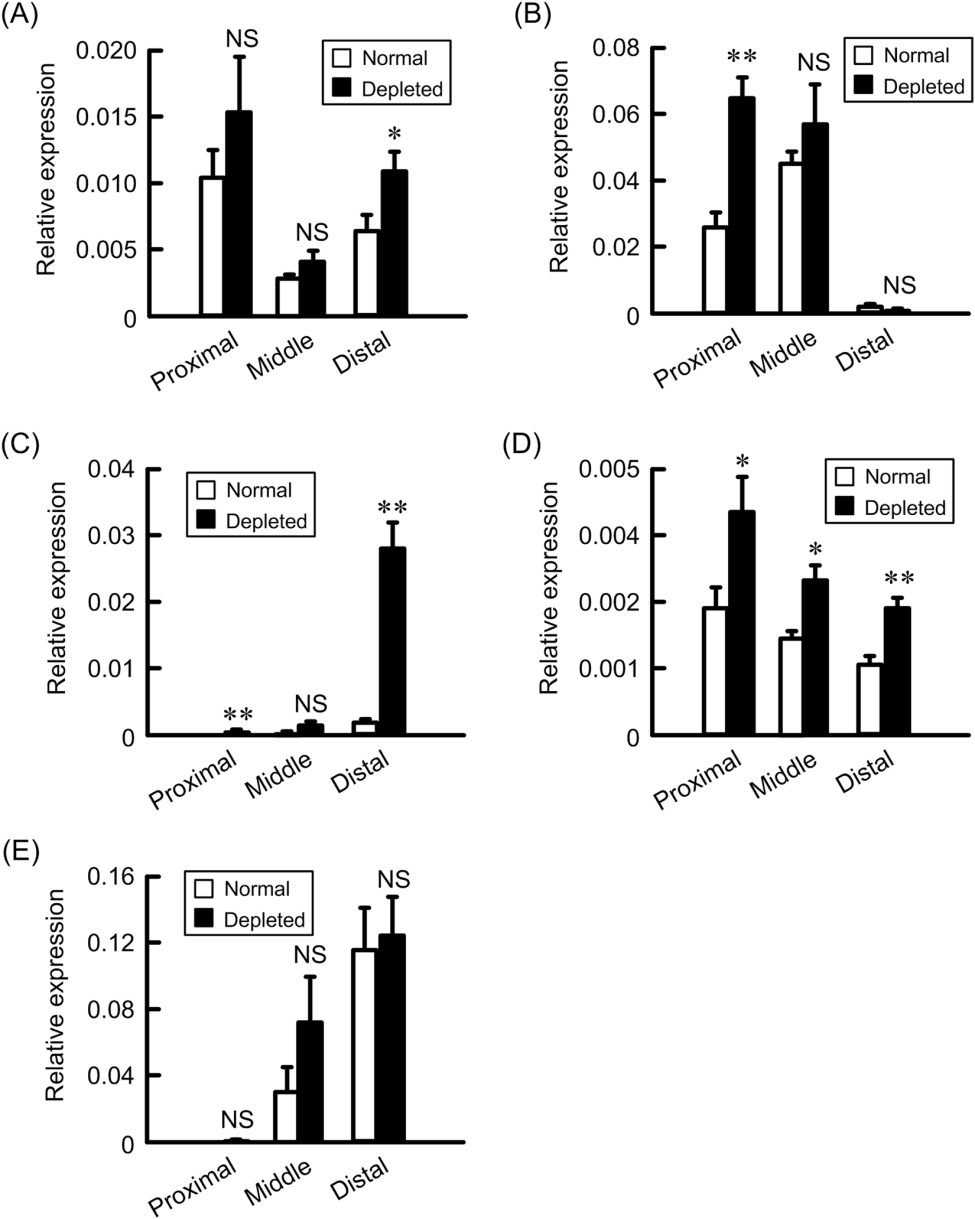
Effect of NaCl depletion on colonic expression of Na^+^ and K^+^ transport systems. The colon was isolated from the mice fed normal and NaCl-depleted diets. After isolation of total RNA, RT-PCR was performed using primers pair of mouse claudin-2 (**A**), NHE3 (**B**), ENaC (**C**), BK channel (**D**), HKA (**E**), and β-actin. The contents of these transporters were represented relative to the values of β-actin. n = 4. Statistical comparison was made by *t* test. **P < 0.01 and *P < 0.05 significantly different from normal. NS, not significantly different.

In the enterocytes, the amount of Na^+^ absorption and K^+^ secretion are regulated by NHE3, ENaC, large-conductance calcium-activated potassium (BK) channel, and H^+^/K^+^-ATPase (HKA)^6^. The mRNA levels of claudin-2 and ENaC was increased in the distal colon by NaCl depletion (Fig. 9). In contrast, those of NHE3 and HKA were not increased in the distal colon and that of BK channel was increased in the proximal, middle, and distal colon. These results indicate that claudin-2 may be functionally related to ENaC and BK channel.

## Discussion

A loss of Na^+^ and circulating blood volume cause a change in renal hemodynamics and/or an increase in ALD secretion from the adrenal cortex^12^. The blood concentration of ALD was elevated in the mice fed with NaCl-depleted diets (Fig. 1D). A compensatory mechanism may be activated during hyponatremia, but the mechanism of action on the colonic TJs has not been fully understood. Paracellular permeability to cations is controlled by claudin-2 and -15 in the small intestine, but the roles of these claudins are slightly different. The knockout mice of claudin-15 show that low concentration of luminal Na^+^ and megaintestine phenotype in the adult intestine^13^. In contrast, that of claudin-2 does not disturb luminal Na^+^ concentration and show slight abnormal structure of villi^14^. The protein levels of colonic claudin-2 were elevated in the mice fed with NaCl-depleted diets, but those of claudin-15 were not (Fig. 1A and B). Claudin-2 was exclusively localized at the crypt epithelial cells in the middle and distal colon under normal diets, and NaCl-depletion increased the expression in the distal colon (Fig. 8). We suggest that claudin-2 may work in not only small intestine, but also distal colonic crypts.

The nutrients including glucose, amino acid, and bile acid are absorbed in the small intestine mediated through Na^+^-dependent systems^15,16^. Double-knockout mice of claudin-2 and -15 lead to infant death from malnutrition^17^, suggesting that both claudins play a critical role in providing paracellular Na^+^ backflow from the blood to lumen. Na^+^ concentration in the gut lumen of mice fed with low NaCl diets is low and the distal colon has a lumen negative potential (mean −26 ± 5 and maximal −45 ± 11 mV)^18^. Therefore, it cannot be assumed that claudin-2 increases Na^+^ absorption, but it will lead to a Na^+^ loss into the colonic lumen. Butyrate, a short chain fatty acid (SCFA), plays a crucial role in colonocyte growth and differentiation^19^. The absorption of SCFA in the colon may be linked with electroneutral NaCl absorption mediated through NHE and Cl^–^butylrate exchanger^20^. Active extrusion of Na^+^ across basolateral membrane is mediated by Na^+^/K^+^-ATPase (NKA), but luminal Na^+^ is required for absorption of SCFA. Interestingly, HKA switches to work as an apical NKA and to actively extrude Na^+^ in parallel with an uptake of K^+^ in the rats fed with low NaCl diets^21^. The mRNA levels of claudin-2 and HKA were elevated in the distal colon of NaCl-depleted mice (Fig. 9). Claudin-2 and HKA may be involved in the supply of luminal Na^+^ in the distal colon under NaCl-depleted diets. Another function of claudin-2 is suggested to control K^+^ secretion. Paracellular K^+^ secretion in the rabbit descending colon has been postulated by Fromm & Schultz^22^. The expression of HKA is up-regulated in rats fed with low Na^+^ diets, which leads to increase in K^+^ absorption. Absorption of SCFA requires luminal H^+^, which is usually supplied by NHE^20^, but the function is inhibited under low Na^+^ conditions. Therefore, H^+^ is actively extruded through HKA, resulting in the promotion of K^+^ absorption. Nevertheless, low Na^+^ diets induce K^+^ secretion in the colon. BK channel is a relevant candidate for colonic K^+^ secretion in the apical membrane. The increment of BK channel in NaCl-depleted diets was observed in all segments of the colon (Fig. 9). The expression of claudin-2 mRNA was significantly increased by NaCl-depleted diets in the distal colon. In addition, dietary NaCl depletion increased claudin-2 expression in the middle part of crypt of distal colon (Fig. 8). BK channel preferentially localizes at the apical membrane of crypt but not surface cells in the distal colon^23^. We suggest that claudin-2 and BK channel may be cooperatively involved in the regulation of K^+^ secretion in the distal colon. Further studies are needed to clarify the physiological role of claudin-2 in the distal colon.

The levels of claudin-7 were also elevated by NaCl-depleted diets. In the knockdown, overexpression, and knockout mice experiments of claudin-7, claudin-7 is shown to function as a Cl^−^ permeable pore in the kidney^24–26^. The expression levels of renal ENaC and Na^+^, Cl^−^ cotransporter are compensatorily increased in the knockout mice. Renal claudin-7 may play an important role in the regulation of Cl^−^ homeostasis. Tanaka *et al*.^27^ reported that colonic claudin-7 forms the paracellular barrier to *N*-formyl-L-methionyl-L-leucyl-L-phenylalanine, a major bacterial product, using the conditional knockout mice. The effect of colonic claudin-7 on permeability to electrolyte ions has not been clarified, but it may be also involved in the Cl^−^ absorption because the expression levels increased in response to NaCl-depleted diets.

ALD increased claudin-2 levels in a dose-dependent manner without affecting those of claudin-4, -7, -8, and -15 (Fig. 4A). Glucocorticoids have been recently reported to decrease claudin-2 expression and increase claudin-4 expression in Caco-2 cells mediated by the phosphorylation of MAPK phosphatase-1^28^. In contrast, the effect of ALD, the principal mineralocorticoid, on the expression of claudins has not been examined in detail. The nuclear localization of MR was increased by ALD (Fig. 4B and C). The cytosolic localization of MR was unchanged by ALD, suggesting that the cytosolic levels of MR are very low under control conditions. The ALD-induced elevation of claudin-2 and nuclear MR was inhibited by spironolactone, but not by RU486 (Fig. 5). This is the first report showing that ALD is involved in the up-regulation of colonic claudin-2. MR with high affinity for binding to ALD is localized to the tissues known as ALD-targeting tissues including colon, parotid glands and distal renal tubule of kidney. In contrast, GR with high affinity for glucocorticoid is expressed ubiquitous. Two distinct molecular mechanisms of ALD action are recognized^29^. One is the classical mechanism involved in the activation and repression via direct interaction of MR with DNA binding sites. Another is the novel mechanism involves transcription interference and synergy via binding of MR with other protein. Two putative SREs were found in the promoter region of human *claudin-2* gene (Supplementary Figure S1) and the reporter activity was inhibited by the mutation in SRE-1. Furthermore, the binding of MR on the promoter region was increased by ALD, which was selectively inhibited by spironolactone. Therefore, we suggest that ALD increases the transcriptional activity of claudin-2 mediated by the classical mechanism.

Claudin-4 and ZO-1 were abundantly expressed in the TJs under control conditions, whereas the expression of claudin-2 was very low in MCE301 cells (Fig. 7). ALD increased the tight junctional localization of claudin-2, which was inhibited by spironolactone. These phenomena are good agreement with the data showing that ALD decreased TER, which was inhibited by spironolactone. The reduction of TER by claudin-2 expression is reported in renal tubular epithelial cells^30,31^. The relation between the reduction of claudin-2 and the elevation of TER is suggested in human colon cancer Caco-2, HT-29, and T84 cells^28,32,33^. Claudin-2 is little expressed in human normal colon and is elevated in inflamed colonic tissues. Claudin-2 expression may be induced by compensatory stimulation of ALD and inflammatory response.

ALD has been reported to increase claudin-8 expression without changing claudin-2 expression using HT-29/B6-GR cells which exogenously express GR^34^. Claudin-8 forms cation selective barrier in renal tubular epithelial cells^35^. Amasheh *et al*.^34^ suggest that claudin-8 has a function of preventing Na^+^ back leakage from plasma to luminal fluid. The activation of MR by ALD increases the expression of claudins-4 and -8, and their localization in the TJs of ALD-sensitive distal nephron^36^. However, our data indicate that ALD did not increase the mRNA and protein levels of claudin-4 and -8 in MCE301 cells. MCE301 cells are derived from the distal colon of transgenic mice. The difference of organ, species and/or GR expression may change sensitivity of claudin-4 and -8 expression in response to ALD.

In conclusion, NaCl-depleted diets increased serum ALD concentration and colonic expression of claudin-2 and -7 in mice. ALD increased the expression level of claudin-2 at the TJs mediated by the nuclear elevation of MR in the murine colonic epithelial MCE301 cells. ALD decreased paracellular permeability to ions, which was inhibited by spironolactone. We suggest that hyponatremia increases ALD secretion from adrenal cortex, resulting in the elevation of Na^+^ absorption mediated through NHE3 and ENaC, and elevation of K^+^ secretion mediated through BK channel. Claudin-2 may have a role in controlling the paracellular transport of Na^+^ and K^+^ in the colon.

## Materials and Methods

### Materials

Anti-claudin-2, claudin-4, claudin-7, claudin-8, claudin-15, and ZO-1 antibodies were obtained from Zymed Laboratories (South San Francisco, CA, USA). Anti-GR antibody was from Bioworld technology (St. Louis Park, MN, USA). Anti-MR antibody was from Bioss (Woburn, MA, USA). Anti-nucleoporin p62 antibody was from Becton Dickinson Biosciences (San Jose, CA, USA). ALD was from Wako Pure Chemical Industries (Osaka, Japan). Spironolactone was from Tokyo Chemical Industry (Tokyo, Japan). RU-486 was from LKT laboratories (St. Paul, MN, USA). All other reagents were of the highest grade of purity available.

### Animals and tissue preparation

Male C57BL/6JJcl mice (8 weeks) were obtained from CLEA Japan SLC (Tokyo, Japan). The mice were randomly divided into two groups; normal diet containing 0.2% Na^+^ and 0.75% K^+^, and Na^+^-free diets containing 0% Na^+^ and 0.75% K^+^, and were provided with food and water ad libitum for 10 days. All animal experiments were approved by the Animal Care and Use Committee of the University of Shizuoka (No. 145080), and conducted in accordance with the Guidelines and Regulations for the Care and Use of Experimental Animals by the University of Shizuoka. The animals were anesthetized with an intraperitoneal injection of a mixture of medetomidine (0.3 mg/kg body weight), midazolam (4 mg/kg body weight) and butorphanol tartrate (5 mg/kg body weight) and a 3 cm segment of mid-distal colon was excised and then opened along the longitudinal axis. The mucosal-submucosal preparation, consisting of the mucosa, muscularis mucosa, and submucosal layers was obtained with fine forceps and frozen by liquid nitrogen. The samples were lysed by 2 x Laemmli sample buffer (125 mM Tris-HCl, pH 6.8, 20% glycerol, 4% SDS, 10% β-mercaptoethanol, and 0.004% bromophenol blue).

### SDS-polyacrylamide gel electrophoresis (SDS-PAGE) and immunoblotting

Nuclear and cytoplasmic extracts were prepared using NE-PER nuclear and cytoplasmic extraction reagents (Thermo Fisher Scientific, Waltham, MA, USA) according to the manufacturer’s instructions. The cytoplasmic extracts include plasma membrane and cytosolic proteins. Samples were applied to SDS-PAGE and blotted onto a polyvinylidene fluoride membrane. The membrane was then incubated with each primary antibody (1:1,000 dilution) at 4 °C for 16 h, followed by a peroxidase-conjugated secondary antibody (1:3,000 dilution) at room temperature for 1.5 h. Finally, the blots were incubated in EzWestLumi plus (ATTO Corporation, Tokyo, Japan) or ImmunoStar Basic (Wako Pure Chemical Industries) and scanned with a C-DiGit Blot Scanner (LI-COR Biotechnology, Lincoln, NE). Band density was quantified with ImageJ software (National Institute of Health software). β-actin or nucleoporin p62 was used for normalization.

### Measurement of serum ALD concentration

Samples of serum were collected from mice fed with normal and NaCl-free diets. The concentration of serum ALD was measured by radioimmunoassay (Oriental Yeast, Tokyo, Japan).

### Cell culture

Mouse colonic MCE301 cell line was established by Tabuchi *et al*.^37^. Cells were grown in Dulbecco’s Modified Eagle’s Medium/Ham’s Nutrient Mixture F-12 (Sigma-Aldrich, St. Louis, MO, USA) supplemented with 10% fetal calf serum, 0.07 mg/ml penicillin-G potassium, and 0.14 mg/ml streptomycin sulfate in a 5% CO_2_ atmosphere at 37 °C.

### RNA isolation and quantitative RT-PCR

The animals were anaesthetized and the whole large intestine was excised and was cut into 3 segments equally in length. Each segment was opened into a flat sheet, and submerged in RNA later for 24 h. The musculature was removed by blunt dissection and the resulting mucosal-submucosal sheet was used for total RNA extraction. Total RNA was extracted using the Nucleospin RNA kit (Macherey-Nagel, Germany). For MCE301 cells, total RNA was isolated using ISOGEN II (NIPPON GENE, Toyama, Japan). Reverse transcription was carried out with ReverTra Ace qPCR RT Kit (Toyobo Life Science, Osaka, Japan). PCR was carried out with DNA Engine Dyad Cycler (Bio-Rad, Hercules, CA, USA) using Go Taq DNA polymerase (Promega, Madison, WI, USA) and primers pairs of mouse claudin-2, -4, -7, -8, -15, and β-actin (Supplementary Table S1). The PCR products were analyzed by agarose gel electrophoresis. Quantitative real time PCR was performed using FastStart Universal SYBR Green Master (Roche Applied Science, Mannheim, Germany). The threshold cycle (Ct) for each PCR product was calculated with the instrument’s software, and Ct values obtained for claudins were normalized by subtracting the Ct values obtained for β-actin. The resulting ΔCt values were then used to calculate the relative change in mRNA expression as a ratio (R) according to the equation R = 2^−(ΔCt(treatment)−ΔCt(control))^.

### TER and paracellular permeability to FD4

Cells were cultured on transwells with polyester membrane inserts (Corning Incorporated-Life Sciences, Acton, MA). TER was measured using a Millicell-ERS epithelial volt-ohmmeter (Millipore, Billerica, MA, USA). TER values (ohms x cm^2^) were normalized by the area of the monolayer and were calculated by subtracting the blank values from the filter and the bathing medium. The paracellular diffusion of dialyzed FD4 (125 μM) for 1 h from the apical-to-basal compartments was measured with a GloMax-Multi + Microplate Multimode Reader (Promega).

### Immunofluorescence

The segments of mouse middle and distal colon were taken and immediately frozen with optimal cutting temperature compound and sectioned (10 µm) on a cryostat. Sections on glass slides were air-dried and incubated in cold methanol for 10 min at −30 °C. Sections were then incubated with a blocking solution of 5% normal donkey serum in phosphate buffered saline (PBS) for 1 h at room temperature. Following pre-blocking, sections were stained with anti-claudin-2 antibody (1:500) for 2 h. After washing, they were incubated with Alexa Fluor 488-conjugated antibody (1:1000) for 1 h. Finally, slides were washed and cover slips were mounted using mounting medium. MCE301 cells were cultured on transwells with polyester membrane inserts for 21 days. The cells were fixed with methanol for 10 min at -20 °C and then permeabilized with 0.2% Triton X-100 for 15 min. After blocking with 2% Block Ace (Dainippon Sumitomo Pharma, Osaka, Japan) for 30 min, the cells were incubated with anti-claudin-2, claudin-4, claudin-7, MR, or ZO-1 antibody (1:100) for 16 h at 4 °C. They were then incubated with Alexa Fluor 488- and 568-conjugated antibodies (1:100) including 4′,6-diamidino-2-phenylindole (DAPI) for 1.5 h at room temperature. Immunolabelled cells were visualized on LSM 700 confocal microscope (Carl Zeiss, Oberkochen, Germany).

### Luciferase reporter assay

The reporter vector of the human *claudin-2* gene was made as described previously^38^. A *Renilla* construct, pRL-TK vector (Promega), was used for normalizing transfection efficiency. Cells were transfected with plasmid DNA using HilyMax (Dojindo laboratories, Kumamoto, Japan). After 48 h of transfection, luciferase activity was assessed using the Dual-Glo Luciferase Assay System (Promega). The luminescence of the *Firefly* and *Renilla* luciferase was measured with an AB-2270 Luminescencer Octa (Atto Corporation). The mutants of putative ALD binding sites (steroid-response elements, SRE-1: -2,021/-2,008 and SRE-2: -1,051/-1,036) were generated using a KOD-Plus-Mutagenesis kit (Toyobo Life Science). The primer pair was described in Supplementary Table S1.

### ChIP assay

Cells were treated with 1% formaldehyde to crosslink the protein to the DNA. ChIP assay was carried out as described previously^38^. To co-immunoprecipitate the DNA, anti-MR antibody and rabbit IgG were used. The eluted DNA was amplified by semi-quantitative PCR. The primers used for PCR are listed in Supplementary Table S2. To confirm the same amounts of chromatins used in immunoprecipitation between groups, input chromatin was also used.

### Statistical analysis

Results are presented as means ± S.E.M. Differences between groups were analyzed with a one-way analysis of variance, and corrections for multiple comparison were made using Tukey’s and Dunnett’s multiple comparison tests. Comparisons between two groups were made using Student’s *t* test. Significant differences were assumed at *p* < 0.05.

## Electronic supplementary material

Supplementary Info
