# Supplementary material for: Up-regulation of claudin-2 expression by aldosterone in colonic epithelial cells of mice fed with NaCl-depleted diets
Source: Sci Rep. 2017 Sep 22;7:12223. doi: 10.1038/s41598-017-12494-1 (PMC5610316; doi:10.1038/s41598-017-12494-1)
Supplement: Supplementary file 1 — Supplementary Info [file 41598_2017_12494_MOESM1_ESM.doc]

Supplementary Information

Up-regulation of claudin-2 expression by aldosterone in colonic epithelial cells of mice fed with NaCl-depleted diets

**Chisa Furukawa^1^, Noriko Ishizuka^2^, Hisayoshi Hayashi^2^, Naoko Fujii^1^, Aya Manabe^1^, Yoshiaki Tabuchi^3^, Toshiyuki Matsunaga^1^, Satoshi Endo^1^, and Akira Ikari^1*^,**

**^1^ From the Laboratory of Biochemistry, Department of Biopharmaceutical Sciences,**

**Gifu Pharmaceutical University, Gifu, Japan**

**^2^ School of Food and Nutritional Sciences, University of Shizuoka, Shizuoka, Japan**

**^3^ Life Science Research Center, University of Toyama, Toyama, Japan**

To whom correspondence should be addressed: Akira Ikari, Ph.D.

Laboratory of Biochemistry,

Department of Biopharmaceutical Sciences,

Gifu Pharmaceutical University,

1-25-4 Daigaku-nishi, Gifu 501-1196, Japan,

Tel: +81-58-230-8124; Fax: +81-58-230-8124

E-mail: ikari@gifu-pu.ac.jp

Supplementary Table S1. Primers for PCR amplification

| Name | Sequence |
| --- | --- |
| Claudin-2 sense | 5’-TGCGACACACAGCACAGGCATCAC-3’ |
| Claudin-2 antisense | 5’-TCAGGAACCAGCGGCGAGTAGAA-3’ |
| Claudin-4 sense | 5’-TCGTGGGTGCTCTGGGGATGCTT-3’ |
| Claudin-4 antisense | 5’-GCGGATGACGTTGTGAGCGGTC-3’ |
| Claudin-7 sense | 5’-GGCCACTCGAGCCTTAATGGTG-3’ |
| Claudin-7 antisense | 5’-CCTGCCCAGCCGATAAAGATGG-3’ |
| Claudin-8 sense | 5’-CATGCCAACATCAGAATGCAGT-3’ |
| Claudin-8 antisense | 5’-CTGTGGTCCAGCCTATGTAGAG-3’ |
| Claudin-15 sense | 5’-GCCTGTGGGATGGTGGCTATCTCGT-3’ |
| Claudin-15 antisense | 5’-TGGTGGCTGGTTCCTCCTTG-3’ |
| β-actin sense | 5’-ccaaccgtgaaaagatgacc-3’ |
| β-actin antisense | 5’-ccagaggcatacagggacag-3’ |
| ALD-M1 sense | 5’-GAATTCCAGGGAACACAGGGAAGAACTG-3’ |
| ALD-M1 antisense | 5’-CTGAATGATACCTCTATCCATCCACT-3’ |
| ALD-M2 sense | 5’-GAATTCGTGTGGGCAACATGGCAAAACC-3’ |
| ALD-M2 antisense | 5’-TGAACTCCTAAGCTCAAGTGATCCACC-3’ |
| NHE3 sense | 5’-ATGTCAGTGCTGTATGCCTGGA-3’ |
| NHE3 antisense | 5’-CGTGCCGACTATAGAGATGCTTG-3’ |
| ENaC sense | 5’-AATGTTCTCACCTGGGACCAAAG-3’ |
| ENaC antisense | 5’-GTCCACCAAAGTTAGACAGGAGCA-3’ |
| BK channel sense | 5’-TTGCTTGTGGGACAGCATTTG-3’ |
| BK channel antisense | 5’-TCCTGTCACCAGGGTCCGTA-3’ |
| HKA sense | 5’-ATCATCGTTGCGTTGGTCCTC-3’ |
| HKA antisense | 5’-TGAACAACTTCCGCATCTCGTC-3’ |

Supplementary Table S2. Primers for PCR amplification of ChIP

| Name | Sequence |
| --- | --- |
| Claudin-2 sense | 5’-GGGATGATGAAACCCTGAATTA-3’ |
| Claudin-2 antisense | 5’-GCAAATGAGAACCCTTACCATC-3’ |


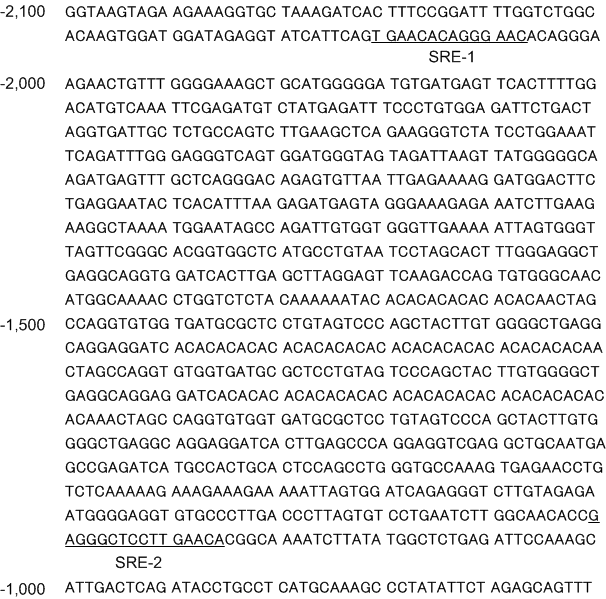


Supplementary Figure S1. The 5'-flanking regions of human claudin-2 from -2,100 to +37 was cloned into the promoter-less pGL4.10 vector. Two putative binding motifs of MR were detected at -2,021/-2,008 and -1,051/-1,036, and are indicated as SRE-1 and SRE-2, respectively.
